# Supplementary material for: Diet drove brain and dental morphological coevolution in strepsirrhine primates
Source: PLoS One. 2022 Jun 6;17(6):e0269041. doi: 10.1371/journal.pone.0269041 (PMC9170099; doi:10.1371/journal.pone.0269041)
Supplement: S1 Table — Description of anatomical landmarks used to capture brain morphology and analogous landmarks from Bertrand et al. [33] and Ahrens [34]. (DOCX) [file pone.0269041.s001.docx]

Table S1. Description of anatomical landmarks used to capture brain morphology and analogous landmarks from Bertrand et al. [33] and Ahrens [34].

| **Number** | **Definition of Landmarks** | **Source** |  |
| --- | --- | --- | --- |
| 1 | Rostral Terminus of Olfactory Bulb | Ahrens (2014) | 1 |
| 2 | Lateral Terminus of Olfactory Bulb | Ahrens (2014) | 2 |
| 3 | Confluence of Circular and Sagittal Fissure | Bertrand et al. (2019) | 3 |
| 4 | Medial-Anterior most end of Olfactory Bulb | Bertrand et al. (2019) | 4 |
| 5 | Dorsal most point on Olfactory Bulb | Ahrens (2014) | 3 |
| 6 | Lateral Constriction of Olfactory Bulb | Ahrens (2014) | 4 |
| 7 | Inferior Fossa of Olfactory Bulb | Bertrand et al. (2019) | 7 |
| 8 | Dorsum Sellae | Bertrand et al. (2019) | 9 |
| 9 | Endobasion | Bertrand et al. (2019) | 10 |
| 10 | Endopisthion | Bertrand et al. (2019) | 11 |
| 11 | Ventral Terminus of Piriform Lobe | Ahrens (2014) | 7 |
| 12 | Rostral-most aspect of the Sylvian Fissure/Fossa | Bertrand et al. (2019) | 14 |
| 13 | Superior most Point on Endocast | Bertrand et al. (2019) | 15 |
| 14 | Internal Occipital Groove | Ahrens (2014) | 14 |
| 15 | Lateral-most Point on Endocast | Bertrand et al. (2019) | 17 |
| 16 | Lateral-most Point on Petrosal Lobule | Ahrens (2014) | 17 |
| 17 | Occipital Pole (Lateral Lobe of the Cerebellum) | Ahrens (2014) | 15 |
| 18 | Caudal-most Point of Occipital (Vermis) | Ahrens (2014) | 13 |
| 19 | Confluence of the Sinsus | Ahrens (2014) | 12 |
| 20 | Ventral-most Point on Petrosal Lobule | Bertrand et al. (2019) | 22 |
| 21 | Dorsal-most Point on Petrosal Lobule | Bertrand et al. (2019) | 23 |
| 22 | Caudal-most Point on Neocortex | Ahrens (2014) | 8 |
| 23 | Ventral-most Point on Neocortex | Bertrand et al. (2019) | 25 |
| 24 | Rostral-most Point on Neocortex | Bertrand et al. (2019) | 26 |
| 25 | Bifurcation of the optic nerves | Ahrens (2014) | 19 |
| 26 | Ventral Terminus of Olfactory Bulbs | Bertrand et al. (2019) | 29 |
| 27 | Rostral-most Point on Petrosal Lobule | Bertrand et al. (2019) | 30 |
| 28 | Caudal-most aspect of the Petrosal Lobule | - | - |
| 29 | Superior-most aspect of the internal acoustic meatus | - | - |
| 30 | Superior-most aspect of the hypoglossal canal | - | - |
