# Supplementary material for: Diet drove brain and dental morphological coevolution in strepsirrhine primates
Source: PLoS One. 2022 Jun 6;17(6):e0269041. doi: 10.1371/journal.pone.0269041 (PMC9170099; doi:10.1371/journal.pone.0269041)
Supplement: S2 Table — Statistical test for differences in per-species evolutionary rates across dietary guilds and pairwise comparisons. (DOCX) [file pone.0269041.s002.docx]

Table S2. Statistical test for differences in per-species evolutionary rates across dietary guilds and pairwise comparisons.

| Trait | Guild | Rate difference | P |
| --- | --- | --- | --- |
| Brain shape | Frugivory | -0.026 | **0.053** |
|  | Insectivory | 0.009 | 0.699 |
|  | Folivory | 0.024 | 0.905 |
|  | Frugivory_Folivory | -0.033 | **0.044** |
|  | Insectivory_Folivory | -0.012 | 0.286 |
|  | Insectivory_Frugivory | 0.021 | 0.856 |
| Relative brain size | Frugivory | -0.015 | 0.087 |
|  | Insectivory | 0.008 | 0.741 |
|  | Folivory | 0.010 | 0.801 |
|  | Frugivory_Folivory | -0.016 | 0.133 |
|  | Insectivory_Folivory | -0.002 | 0.432 |
|  | Insectivory_Frugivory | 0.014 | 0.857 |
| Dental morphology | Frugivory | -0.317 | 0.325 |
|  | Insectivory | 0.520 | 0.786 |
|  | Folivory | -0.163 | 0.489 |
|  | Frugivory_Folivory | -0.052 | 0.394 |
|  | Insectivory_Folivory | 0.486 | 0.775 |
|  | Insectivory_Frugivory | 0.538 | 0.798 |
|  |  |  |  |
