# Supplementary material for: Diet drove brain and dental morphological coevolution in strepsirrhine primates
Source: PLoS One. 2022 Jun 6;17(6):e0269041. doi: 10.1371/journal.pone.0269041 (PMC9170099; doi:10.1371/journal.pone.0269041)
Supplement: S3 Table — Statistical test for differences in per-guild evolutionary rates across dietary guilds. Net evolutionary rates are provided per guild for each trait. (DOCX) [file pone.0269041.s003.docx]

Table S3. Statistical test for differences in per-guild evolutionary rates across dietary guilds. Net evolutionary rates are provided per guild for each trait.

|  | K | Z | P | Folivory | Frugivory | Insectivory |  |
| --- | --- | --- | --- | --- | --- | --- | --- |
| Relative brain size | 2.908 | -0.071 | 0.530 | 0.015 | 0.006 | 0.017 | |
| Brain shape | 1.742 | 1.357 | **0.096** | 0.004 | 0.002 | 0.003 | |
| Dental morphology | 2.379 | -0.029 | 0.507 | 0.789 | 0.647 | 1.539 | |
