# Supplementary material for: Diet drove brain and dental morphological coevolution in strepsirrhine primates
Source: PLoS One. 2022 Jun 6;17(6):e0269041. doi: 10.1371/journal.pone.0269041 (PMC9170099; doi:10.1371/journal.pone.0269041)
Supplement: S4 Table — Significance values for pairwise statistical tests of differences in per-guild evolutionary rates across traits. (DOCX) [file pone.0269041.s004.docx]

Table S4. Significance values for pairwise statistical tests of differences in per-guild evolutionary rates across traits.

| Brain shape |  |  |  |
| --- | --- | --- | --- |
|  |  | Folivory | Frugivory |
|  | Frugivory | **0.035** |  |
|  | Insectivory | 0.448 | 0.187 |
| Brain size |  |  |  |
|  |  | Folivory | Frugivory |
|  | Frugivory | 0.358 |  |
|  | Insectivory | 0.867 | 0.238 |
| Dental morphology |  |  |  |
|  |  | Folivory | Frugivory |
|  | Frugivory | 0.818 |  |
|  | Insectivory | 0.428 | 0.213 |
