# Supplementary material for: Diet drove brain and dental morphological coevolution in strepsirrhine primates
Source: PLoS One. 2022 Jun 6;17(6):e0269041. doi: 10.1371/journal.pone.0269041 (PMC9170099; doi:10.1371/journal.pone.0269041)
Supplement: S5 Table — Test of phylogenetic signal in per-species evolutionary rates. (DOCX) [file pone.0269041.s005.docx]

Table S5. Test of phylogenetic signal in per-species evolutionary rates.

|  | K | Z | P |
| --- | --- | --- | --- |
| Relative brain size | 0.699 | 1.723 | **0.044** |
| Brain shape | 0.677 | 1.513 | 0.068 |
| Dental morphology | 1.567 | 2.183 | **0.001** |
