# Supplementary material for: Diet drove brain and dental morphological coevolution in strepsirrhine primates
Source: PLoS One. 2022 Jun 6;17(6):e0269041. doi: 10.1371/journal.pone.0269041 (PMC9170099; doi:10.1371/journal.pone.0269041)
Supplement: S1 File — (DOCX) [file pone.0269041.s007.docx]

**REPLICATED RESULTS WITHOUT AYE-AYE**

Procrustes ANOVAs testing for differences in allometry- and phylogeny-corrected brain size and shape and dental morphology across dietary guilds (see Table 3 for results with full sample).

|  | Df | SS | MS | R^2^ | F | Z | Pr(>F) |
| --- | --- | --- | --- | --- | --- | --- | --- |
| Brain shape | 2 | 0.076 | 0.038 | 0.180 | 1.751 | 2.326 | **0.012** |
| Brain size | 2 | 0.031 | 0.016 | 0.251 | 2.685 | 1.302 | **0.098** |
| Dental morphology | 2 | 1.331 | 0.666 | 0.456 | 6.713 | 2.684 | **0.002** |

Statistical test for differences in per-species evolutionary rates across dietary guilds and pairwise comparisons (see S2 Table for results with full sample).

| Trait | Guild | Rate difference | P |
| --- | --- | --- | --- |
| Brain shape | Frugivory | -0.022 | **0.067** |
|  | Insectivory | -0.003 | 0.428 |
|  | Folivory | 0.031 | 0.975 |
|  | Frugivory_Folivory | -0.034 | **0.02** |
|  | Insectivory_Folivory | -0.025 | 0.088 |
|  | Insectivory_Frugivory | 0.009 | 0.671 |
| Relative brain size | Frugivory | -0.010 | 0.19 |
|  | Insectivory | 0.002 | 0.603 |
|  | Folivory | 0.011 | 0.805 |
|  | Frugivory_Folivory | -0.013 | 0.166 |
|  | Insectivory_Folivory | -0.006 | 0.365 |
|  | Insectivory_Frugivory | 0.007 | 0.722 |
| Dental morphology | Frugivory | 0.007 | 0.499 |
|  | Insectivory | -0.177 | 0.272 |
|  | Folivory | 0.168 | 0.756 |
|  | Frugivory_Folivory | -0.120 | 0.315 |
|  | Insectivory_Folivory | -0.254 | 0.21 |
|  | Insectivory_Frugivory | -0.134 | 0.344 |

Statistical test for differences in per-guild evolutionary rates across dietary guilds. Net evolutionary rates are provided per guild for each trait (see S3 Table for results with full sample).

|  | K | Z | P | Folivory | Frugivory | Insectivory |
| --- | --- | --- | --- | --- | --- | --- |
| Relative brain size | 3.080 | -0.075 | 0.532 | 0.013 | 0.006 | 0.020 |
| Brain shape | 1.737 | 1.051 | 0.160 | 0.004 | 0.002 | 0.003 |
| Dental morphology | 1.419 | -1.186 | 0.866 | 0.743 | 0.583 | 0.826 |

Significance values for pairwise statistical tests of differences in per-guild evolutionary rates across traits (see S4 Table for results with full sample).

| Brain shape |  |  |  |
| --- | --- | --- | --- |
|  |  | Folivory | Frugivory |
|  | Frugivory | **0.055** |  |
|  | Insectivory | 0.380 | 0.379 |
| Brain size |  |  |  |
|  |  | Folivory | Frugivory |
|  | Frugivory | 0.449 |  |
|  | Insectivory | 0.762 | 0.247 |
| Dental morphology |  |  |  |
|  |  | Folivory | Frugivory |
|  | Frugivory | 0.694 |  |
|  | Insectivory | 0.885 | 0.582 |

Phylogenetic two-block least square analyses for integration in evolutionary rates between brain size and shape and dental morphology (see Table 3 for results with full sample).

|  | R^2^ | Z | P |
| --- | --- | --- | --- |
| Brain shape_ brain size | 0.041 | 0.196 | 0.870 |
| Brain shape_Dental morphology | 0.460 | 1.899 | **0.048** |
| Brain size_Dental morphology | 0.239 | 1.002 | 0.329 |
