# Supplementary material for: Diet drove brain and dental morphological coevolution in strepsirrhine primates
Source: PLoS One. 2022 Jun 6;17(6):e0269041. doi: 10.1371/journal.pone.0269041 (PMC9170099; doi:10.1371/journal.pone.0269041)
Supplement: Attachment — Submitted filename: Review for Lopez-Aguirre.pdf [file pone.0269041.s011.pdf]

Review for: Lopez-Aguirre et al. “Diet drove brain and dental morphological coevolution in stepsirrhine primates”

Do I think this paper should be published? No, not really. I will enumerate the many reasons below. However, rather than trying to block this paper from making it into print, I am going to recommend—counter to my own opinion of the work—that this paper is accepted, I would say without revision, but I leave it up to the authors to revise it as they please and resubmit. I promise to accept without further revision, or to work with the authors until either there is something publishable (which I doubt) or they feel they’ve made enough of an effort that this deserves to be in print (its really up to them). First, I will explain why I am taking this counter-intuitive tact. Then I will explain why this paper is a misadventure in P-Hacking.

Why I am recommend accepting without revision (or with as much revision as they authors want to do):
My decision to recommend accepting this paper without revision (or up to the amount of revising as the authors want to do) is more accurately a reflection of my cynicism about scientific publishing and the peer-review process, than it is about the scientific merit of this paper in particular. Clearly, these authors are motivated to see this into print. They have done a considerable amount of work and must have funds set aside for publishing this paper since they have submitted it to PLoS ONE. Additionally, like everyone else in modern academics, these authors face considerable pressure to make sure their names consistently appear in print. Hiring opportunities, promotions, peer-recognition, even success of grant applications are all predicated on publication volume much more so than publication quality. This is not to say that publication quality is irrelevant to authors or even to these authors of this paper. Its just that we all understand that volume is the far more sought-after signifier of scholarly productivity. The need to publish, the spent effort, and the set aside funds all tell me that if this paper were to be rejected on the basis of my review, the authors would very likely repackage the paper for acceptance to another journal. I’d prefer not to see that happen if this paper makes it into print, but ultimately, I don’t really care that much.

The premise and conclusions of this paper are not controversial. The authors are operating under the idea that brain size and shape is correlated (somehow) with diet, and that dental morphology is also correlated with diet. Thus, dental morphology and brain size/shape are likely epiphenomenally correlated, with diet as the intermediary link. A good deal of research has gone into the investigation of diet-brain links, as partially reviewed by these authors. Yes, as brain size goes up you need more energy to support that tissue and typically it seems dietary quality (i.e. energy density) rises also. Dental morphology has been exhaustively studied and is known to adapt to dietary content, which of course is going to be related to dietary quality. So, when the authors deliver us a significant correlation between these two variables (brains and teeth) no one is going to think twice provided they weave the appropriate tale about their chosen variables. They are also not taking a controversial stance in explaining the correlation. For example, these authors are not arguing that there is a gene or suite of genes controlling *both* brains and teeth, thereby structurally linking these biological aspects of morphology. That would require some interesting evidence and investigation. I’m sure that if pressed, the authors

would agree that there are likely wholly separate gene-clusters responsible for dental and brain form, thus there's no *intrinsic* link between these variables. No, rather the authors don't really provide a mechanism for how these two traits are tracking one-another—other than suggesting they are mutually linked through diet. This is presumably because other than offering the diet narrative, they don't exactly know how these traits are linked, especially given the myriad of abstractions they have applied to their original measurements.

As a statistically literate reviewer, I can see and understand what they've done here. I am not intimated by the procedures or mathematical components of this 'analysis'. However, I am quite aware that not many other reviewers have this particular acumen. This is not any one person or group's fault, of course. For researchers in a broad and complex discipline—such as evolutionary biology—to make meaningful contributions to ongoing debates, they must often specialize in particular areas and on certain types of skills leaving the other skills and knowledge areas underdeveloped. Thus, if I were to recommend rejection of this paper and these well motivated authors were to repackage for another journal, I am unlikely to have this paper fall into my reviewing lap again. Indeed, I estimate that it is unlikely that someone with the technical expertise and confidence necessary will be asked to review this work and see it properly rejected. And so, it will inevitably skate-by eventually sliding into print. Defeating the entire point of peer-review, but this is not new, this has been going on for a long time, and this is not unique to these authors.

Let's assume this paper is rejected by PLoS ONE, and ends up in print elsewhere (or lets also speculate that these authors may opt to withdraw this paper after reading this review, and repackage for elsewhere hoping to avoid me as a reviewer). Why would that be an undesirable outcome from my perspective? Well, I doubt that once in print any serious researcher would spend their time writing a rebuttal. Their conclusions and premise are simply not controversial enough to warrant further attention. This work is not meaningfully driving our understanding of any of the features or phenomena purportedly examined in this paper. Imagine bothering yourself with responding to this paper by submitting an article to PLoS ONE, which would then have to be reviewed by not just other anonymous scholars, but then the original authors of this work would be invited to respond as well. Don't we all have our own research to do without being side-tracked correcting someone else's? And to what end? The specific issues raised, and problems associated with this paper are simply not important enough to do a rebuttal. I don't have the time to do this dance, and no one is going to learn anything from that kind of exchange. Huge waste of time.

So lets be Solomon and offer to cut the baby in half and leave it up to the mothers to decide what they want to do. Given everything stated above, especially the impossibility of blocking this from appearing in print, one redeeming aspect of PLoS ONE makes it worthwhile to recommend accepting this paper without revision (or as much revision as the authors wish); PLoS ONE publishes the anonymous reviewer comments as supplemental material alongside this paper. So, while I am uncertain that anything I say in my review will transform this into meaningful work; and it seems wrong for it to be my job to either make this publishable or write a correction afterward; at least these comments will be permanently appended to this

publication for skeptical readers to peruse after attempting to digest the premise, methods, and results of this paper.

Problems with this paper:
Below I document the many problems with this paper, but first I have some thoughts and caveats.

If I were to summarize this research, I would say: the authors tested for a link between brain size/shape and dental morphology. To do this, they collected landmark data on 'endoscans' as their measure of brain size/shape, and collected measures of dental topography as their stand-in for tooth morphology. The authors then proceeded to 'correct' these data for allometry and phylogeny (incorrectly) and then used the 'corrected' data in further phylogenetically oriented analyses. Specifically, they used the 'corrected' measures to reconstruct evolutionary rates throughout their sample phylogeny, and then used a sophisticated integration tool (also incorrectly) to test to see if the rates of evolution were 'integrated.' Their findings offer some weak *P*-values—which may be entirely driven by Aye-Ayes—and they conclude that Brains and Teeth are ecomorphologically linked. OK. Fine.

Whenever I am pursuing a particular analysis or set of analyses, I like to keep a few basics in mind. Statistical procedures are not all that different from physical tools like a microscope. They are used to help 'see' things (patterns) which may not otherwise be observable because of confounding variables, incomplete data, use of measures which approximate some other un-measurable variable, etc. They are also useful for confirming or refuting the significance of readily observable patterns. Regardless of precisely how/why the analytic procedures are being used, the end goal still remains to provide meaningful insight on the morphology, ecology, and evolution of the study taxa. In this way, the authors will need to translate useful and relevant information from their results. Since the end goal is constant regardless of the particular statistical work-flow or measures, I operate under two complementary rules of thumb: the more complex the measurements I need to use, the *simpler* the analysis I will perform on those measures. AND (the corollary) the more complex the analyses I want to perform, the *simpler* the measurements I will choose to use. Keeping one or the other—the measurement or the analysis—neatly grounded to interpretable reality if often essential for being able to translate useful information from the results. These authors clearly have not pursued this work with my more cautious personal 'rules', I suppose their motivation to publish is over-powering their desired to understand what they've actually measured and analyzed. Or, perhaps they have a deeper understanding of these techniques and measurements than I do and are using these tools in new and creative ways, but I suspect this is not the case.

The package geomorph is an amazing tool and its authors and maintainers have well-earned reputations for doing good and meaningful work. I do not object to any one particular analysis the authors of this paper have performed. Rather, it is the strange and unnecessary over-application of various analytic tools, as well as the use of the derived outputs from those tools

in further downstream analyses which makes this work a P-hacking fiasco. Let's review the obvious flaws in their study design:

- Direct quote from authors: "To elucidate diet-driven ecomorphological patterns, we first quantified the effects of allometry and the presence of phylogenetic structuring in our data. Dental, CS [brain size] and brain shape allometry were tested with a Procrustes regression (using log-transformed BdM [body mass]), implemented in the procD.lm function in the R package Geomorph (39). For CS and brain shape, the residuals of the Procrustes regression were used as allometry-corrected data in all further analyses."
    - Firstly, using residuals does not 'correct' for allometry. Using the residuals may permit examination of other variables which may be swamped-out by the size signal in an analysis. But the allometric effects on shape are not 'corrected' by using these residuals. The shape information remains.
        - Lets illustrate this point with a simple example. Imagine you have three taxa. Imagine you sample size (of some element) and shape (somehow for the same element) among the adult forms of these three taxa. You plot their slopes which represent the allometric relationship (i.e., between size and shape) *within* each taxa. Are they all perfectly aligned among the three unique plots for each taxa? Is one or more of the slopes different among the three taxa? Is one or more of the regression lines up or down shifted from the others? If the answer is yes to any of these latter questions—and you know at least one of those is going to happen—then this demonstrates that each of these taxa or at least one of the many taxa in the analysis has a different allometric line of best fit from the others, suggesting separate allometric relationships. Thus, you can't possibly 'correct for allometry' with a clade-wide sampling of species if there are heterogenous allometric slopes. That's probably why we haven't come up with the "Planck's constant" of allometry and we all resort to doing this residual analysis unique to each paper.
        - This means that no matter where you go from here, this residual is an abstraction and you should be quite careful about its use in downstream analyses unless you can specifically account for what they mean in the morphology or size or what have you.
    - Second, I agree that it is important to test for these features, but many of the functions in Geomorph and the other packages these authors use, enable them to include the confounding variables of unevenly shared historical contingency, and allometry in the analyses directly, rather than using the 'corrected' variables as initial inputs.
    - Finally, I would like to add that I don't think the residuals from such an analysis contain no information. They might hold important information about allometric proportioning, but it is not a very precise estimate of whatever information it is assessing. So anyone using residuals in further analyses needs to be extraordinarily cautious since they are only rough ballpark values—at best—of

whatever proportioning is being measured, and I would discourage their use in any more complex and abstract analysis.

- Dental Morphology: The authors have decided to use Dental Topography measurements to quantify the morphology of their dental sample. These measures are much in vogue at the moment. But it is important to emphasize some facts about these measurements:
    - All of these measurements are landmark-free abstractions of the actual dental morphology. Importantly, while this might not always be how these tools are used, the developers of these methods intended them to be used to capture *functional* properties of the teeth. Thus, these measures ought to be tracking dental functional *performance*. There is some debate as to how well OPCR is actually doing this, particularly within primates (Pineda-Munoz et al. 2017). It seems like the OPCR measurement is unlikely to provide anything insightful about these primate teeth. It works best on rodent and some carnivore teeth with interdigitating lophs on the molars, in fact that was its original application. It does not work well on generalized bunodont primate teeth. DNE and RFI seem to be on better footing, but scholars are still trying to decide what exactly RFI might be tracking. DNE seems to be a proxy for sharpness and might be the most effective measure of the group. So there is certainly an argument to be made that the inclusion of OPCR here is just adding noise to their analysis.
    - Importantly, because these measurements are morphological abstractions only concerned with homology-free quantifications of the surface properties, this means that identical DNE, RFI, and OPCR values can be achieved via quite disparate morphologies. What is the likelihood that 2 totally distinct morphologies which independently arrived on two identical DNE values did so with the same evolutionary tempo? I would say quite low… yet their analysis is blind to this possibility because they haven't accounted for the *morphology* only the abstract estimates of the functional properties of the morphology. That is, by using these measurements they are already 1-step removed from the morphology, a pretty large and abstract step, and they should be very cautious about any further abstractions or complex analyses.
    - Additionally, for some reason that I cannot understand, the authors thought it best to subject their small dental morphology data matrix to a phylogenetically controlled-PCA. They state that this is to "control for phylogenetic structuring and reduce the dimensions of our data…" they continue "…the first three principal components (accounting for 100% of variation) were used for dental morphology in all further analyses."
        - First, they don't reduce their data dimensions for the dental morphology by doing this. They start with three columns of data, they end with three columns of data. So I assume they are only doing this to scrub out the phylogenetic signal.
        - Second, the dental morphology data were *already* abstractions of the true morphology (as noted above), with the specific aim of capturing functional properties of the teeth and yet, the authors will use the principal components of a phylo-PCA in further analyses? What hope

        does anyone have of being able to interpret what PC1 in such an output is morphologically tracking with?
    - Lastly, the authors seem motivated to apply this phylo-PCA because they think it important to "correct for phylogeny" for their subsequent rates analyses. As noted below, that is inappropriate. There are many built-in phylogeny controls in the subsequent methods. No need to do a 'pre-control' on the 'raw data' (which is far from raw).

- "Given the significant phylogenetic signal and multidimensionality of our dental morphology and brain shape data, we performed a phylogenetic principal component analysis (pPCA) on the correlation matrix of each data set to control for phylogenetic structuring and reduce the dimensions of our data, using the gm.prcomp function in Geomorph."
  - While reading over their methods section on the rates analysis, it is unclear to me whether or not they have used the 'phylogenetically corrected' data for these analyses. If they did, this is not a correct application of this technique. All rates-based analyses are based on the fact that there *is* phylogenetic information in the data. It will use the proposed tree to try to reproduce the values at the tips of the tree, first using a single clade-wide rate, then making successive adjustments to the rates of different branches, first inserting one location for a rate-shift, if the fit with the tip data is still poor, another rate-shift will be added somewhere in the phylogeny, UNTIL the trait values at the tips can be well approximated by the model all the while trying to minimize the number of variables in the model (i.e., number of rate-shifts) all the while incorporating the structure of the phylogeny. There are of course slight tweaks and variations that the different rates-estimating functions do. For instance in Geomorph their rates analyses incorporates factor-based partitions which will test to see if clade members in different ecological (in this case diet categories) experience different rates of evolution in the trait in question. This is to say, if the authors scrub the data of phylogenetic signal, then these methods will be reconstructing the production of noise, rather than anything meaningful about the morphology, since these rates analyses are explicitly reliant on using the phylogenetic information contained in the data.
- Another statement in the methods really caught my eye: "We investigated the integration in evolutionary rates between brain and dental morphology using phylogenetic two-block partial least square analysis, accounting for phylogenetic nonindependence in rates of evolution across species, as implemented in the phylo.integration function in Geomorph.
  - I am not sure I read this correctly, but I think the authors performed a test of integration (i.e., modularity) on the reconstructed evolutionary rates for some phylogenetic PCA components representing Brain Size, Brain Shape, and Dental Topography. I don't think this could possibly result in any insights. Firstly, the phylo.integration function is a very specific tool, meant to test to see if two or more sets of coordinates (i.e., original shape data) are integrated together, using a phylogenetic framework. I don't see how this can be properly applied to testing

if the rates throughout a phylogeny for two different models of trait evolution are 'integrated' and I am not sure what that would mean if it were true.

- A much simpler approach would be to extract the branch-rate values from each analysis and then perform a simple regression. This is preferred since it would be correlating specific branches with the like branch in the other model, as opposed to allowing the integration calculations to see if changes occur in both sets of data in a roughly coordinated way. For instance, the integration calculation does not care if the rates are negatively correlated, or positively correlated, its not really capable of telling the difference. Whereas the regression will be able to tell the difference.
- The authors are engaging in way too many abstractions of this data here, not only is it hard to follow what they have done, but I do not trust their ability to correctly interpret these findings into biologically meaningful phenomena.

- The authors note in their Data availability statement that: "All raw data and code needed to replicate analyses will be freely available upon acceptance as supplementary material."
  - I think seeing the code will help me understand what kinds of analyses they have done. I would prefer to see it before publication, but I understand their reluctance.
  - Since the authors are currently withholding their data, I cannot confirm this suspicion I have, but there seems to be good evidence to suggest that if they were to re-perform their elaborate analyses without the inclusion of Aye-Ayes, that their weakly significant results would evaporate. Based on looking at their figures, particularly the 3D scatter plot in Fig. 1, as well as the outlier-ish DNE insectivore box plot 'dot' way below the other insectivore values, as well as the rate plots of Figure 2, it seems safe to say that Aye-Ayes are driving their findings.
    - What a flimsy linchpin for this work. The DNE value certainly doesn't suggest that Aye-Ayes are truly insectivores, causing them to be a near total 'break' from the rest of the clade (I am not saying that the number is wrong, just that there is not the expected correspondence between the value and the ecology, yet the authors don't seem bothered by this). They are clearly driving the result of the Figure 3 scatter plot.
  - And look at the P-values the authors are hanging their hat on in the first paragraph of the results:
    - "After accounting for allometry and phylogenetic structuring, brain shape and dental morphology are significantly different between dietary guilds at $P < 0.03$ [also why not just report the value… what is it? 0.029? that would look better than that!] and relative brain size nearing significance at $P = 0.07$ (Fig. 1 and Table 3)… …Dental sharpness (DNE) and crown height (RFI) are lower in frugivore species and higher in insectivore species, whereas OPCR is higher in folivores and lower in frugivores. Statistically significant morphological integration between dental morphology and both brain size (R^2=0.896, P=0.028) and shape

(R^2=0.795, *P*=0.028) but not between brain size and shape (R^2=0.896, *P*=0.197) signal correlated brain-dental variation and decoupled from brain size and shape variation."

- If Aye-Ayes are removed… will these 'significant' results hold up? I kinda doubt it.

- Is it fair to call Aye-Ayes outliers? I think so, which is why I would expect that the authors need to redo all these analyses with them excluded. I do not doubt the original measurements taken from the Aye-Ayes. I'm sure they are accurate, and so I don't think it's about measurement error. But even so, Aye-Ayes are *biological* outliers. The authors even acknowledge this in their introduction, noting: "a remarkable example of the strepsirrhine diversification is the Aye-Aye (*Daubentonia madagascariensis*) and its combination of morphological traits (e.g., ever-growing incisors, elongated middle digit and squirrel-like skull) associated to its unique percussive foraging strategy." This indicates to me that the authors understand that they should not allow the Aye-Aye data to be the determinant of their results. Run all the analyses a second time with them removed.

- The authors also say: "For each trait (dental morphology, CS [brain size] and brain shape), we fitted three evolutionary models: BM that assumes a random-walk process, Early Burst (EB) assumes rapid phenotypic diversification followed by a decline across time (i.e. adaptive radiation) and Ornstein-Uhlenbeck (OU) that assumes evolution under stabilising selection. Sample-size correct Akaike information criteria (AICc) was used to select the best-supported models, with models with AICc below 2 considered as supported. This procedure was perform for each dietary guild separately."
  - Adams and Collyer (2018) demonstrated that using these methods on multivariate data sets (e.g., dental morphology and brain shape), specifically using mvMORPH, could produce unreliable results. As they wrote: "model evaluation using test measures such as LRT and AIC scores, when based on unstable logL estimates, do not provide a general analytical solution for the evaluation of phylogenetic comparative trends in high-dimensional multivariate data sets." Pg. 20
  - Given the thorough demonstration of the instability of these methods by Adams and Collyer, it should be incumbent upon the authors to demonstrate that their results are reliable in the present case. If they can't, then these analyses should be discarded. Additionally, they have compounded their problem here using not only multivariate, but also highly abstract multivariate reconstructions of rates. Extreme caution should be used in trying to interpret anything for any results coming from these analysis.
